# Viscosity of liquid Co–Sn alloys: thermodynamic evaluation and experiment

**DOI:** 10.1080/00319104.2013.876639

**Published:** 2014-02-05

**Authors:** Andriy Yakymovych, Yuriy Plevachuk, Stepan Mudry, Jürgen Brillo, Hidekazu Kobatake, Herbert Ipser

**Affiliations:** ^a^ Department of Inorganic Chemistry (Materials Chemistry), University of Vienna, Währinger Str. 42, 1090Vienna, Austria; ^b^ Department of Metal Physics, Ivan Franko National University, Kyrylo and Mephodiy str. 8, 79005Lviv, Ukraine; ^c^ Deutsches Zentrum für Luft- und Raumfahrt (DLR), Institut für Materialphysik im Weltraum, 51170Köln, Germany

**Keywords:** Co–Sn, liquid binary alloys, thermodynamic models, viscosity

## Abstract

Shear viscosity measurements were performed for liquid Co–Sn alloys over a wide temperature range above the respective liquidus temperatures. A high temperature oscillating-cup viscometer was used. It was found experimentally that viscosity as a function of temperature obeys an Arrhenius law. The data were compared with calculated values, obtained from different thermodynamic approaches. A good agreement was found between experimental results and calculated ones by the Budai–Benkö–Kaptay model.

## 1. Introduction

Viscosity is one of the most important transport properties of materials in the liquid state. Due to its high sensitivity to structure and phase transformations, the temperature and concentration changes of viscosity can provide exhaustive information about different structural changes, especially those occurring above the liquidus temperature [1,2]. From the practical point of view, viscosity is one of the key parameters for design and optimisation of metallurgical processes.

Sn-based alloys are widely applied in various branches of industry. Due to their ability to form the lithium rich phase Li_4.4_Sn, tin containing alloys are prospective anode materials for rechargeable lithium batteries [3–5]. Tin, as the active component of the electrode, reacts with lithium, while the inactive component (Co, Cu, Fe, etc.) provides a matrix to reduce the massive volume change during the lithiation/delithiation process. The reaction of lithium with Co–Sn alloys leads to the formation of disordered Li_4.4_Sn alloys and nanoscopic grains of cobalt. The reason of using intermetallic Co–Sn compounds is the reversible mechanism, in which nanosized cobalt forms. Such type materials act as a spectator upon the lithiation/delithiation reaction and supports also electronic contact in the material for better cycling performance. The Co–Sn alloys can also be used in specific high-tech applications for devices, chip interconnections and packaging [6]. The replacement of the chromium electroplating by deposited Co–Sn alloys could help solving the problem with the toxicity of chromium.

Co–Sn belongs to the compound-forming systems with the existence of several intermetallic phases in the diagram [7]. Numerous studies of structural [8–10] and thermodynamic properties [11–14] of this system have been performed in both, the liquid and solid state. The preferable interaction between unlike kind atoms for the Co–Sn system is displayed in negative values of the enthalpy of mixing, structure studies in the solid as well as liquid state. Taking into account this type of interatomic interaction, Ivanov [15] calculated the enthalpy of mixing for liquid Co–Sn alloys based on the association theory.

To our knowledge, the viscosity, in contrast to the above-mentioned properties, has not yet been studied for liquid Co–Sn alloys. In general, most transport properties, such as the diffusion coefficient or the electrical conductivity have been studied for Co–Sn either in the solid state [16,17] or using various models [18,19]. The changes of the electrical conductivity of liquid tin upon adding small amounts of cobalt were experimentally investigated in Reference [20] and the diffusion coefficient was studied in Reference [21].

The main goal of the present study is the investigation of the temperature and concentration dependencies of the viscosity over the entire concentration range. The existence of an inhomogeneous short-range order in the liquid should also be displayed in the concentration dependence of the viscosity. The obtained experimental data are compared with thermodynamic models, calculated from different approaches [22–25] at a constant temperature of 1773 K, as well as with literature data.

## 2. Experimental

The viscosity measurements were carried out using a high temperature oscillating-cup viscometer [26]. In this method, a cylindrical alumina crucible containing the sample is placed in a graphite container which is attached to the torsion wire inside the high temperature furnace. The experiments were performed in an atmosphere of argon (400 mbar) after initially evacuating the working volume of the furnace down to a pressure of approximately 10^−8^ mbar. The temperature was estimated by a pyrometer looking at the bottom of the graphite crucible container which has an emissivity close to unity. In addition, the pyrometer was calibrated at the melting temperatures of several pure materials (Al, NaCl, Ag, Cu, Ni and Fe). Samples were prepared by initially melting accurately weighed (within ±0.1 mg) amounts of the pure components Co (99.9+ % metallic purity, Alfa Aesar, USA) and Sn (99.98% metallic purity, Alfa Aesar, USA) in the furnace, which was heated up to 1873 K. This temperature was kept for at least one hour allowing the samples to homogenise. The viscosity was measured during cooling from highest temperature to the liquidus point with a constant cooling rate of 2 K min^−1^.

To study the viscosity of Sn-rich samples with liquidus temperatures, lower than the values, which could be measured by the pyrometer, another oscillating-cup viscometer was used [27]. The temperature was determined by WRe-5/20 thermocouples. All other experimental conditions were almost the same in both experimental sets, and the viscosity was determined with an accuracy of about 5%.

After the measurement, the weight of each sample was checked. In all cases, the loss of material by vaporisation was lower than 0.2% of the ingot.

Using the modified Roscoe equation, the dynamic viscosity, *η*, was calculated from the logarithmic decrement and the period of oscillations [28]. The required density values were calculated for the melts from the densities of the constituents, *ρ_i_*, under the assumption that the excess volume is zero [29].

## 3. Results and discussion

**Figure 1.**
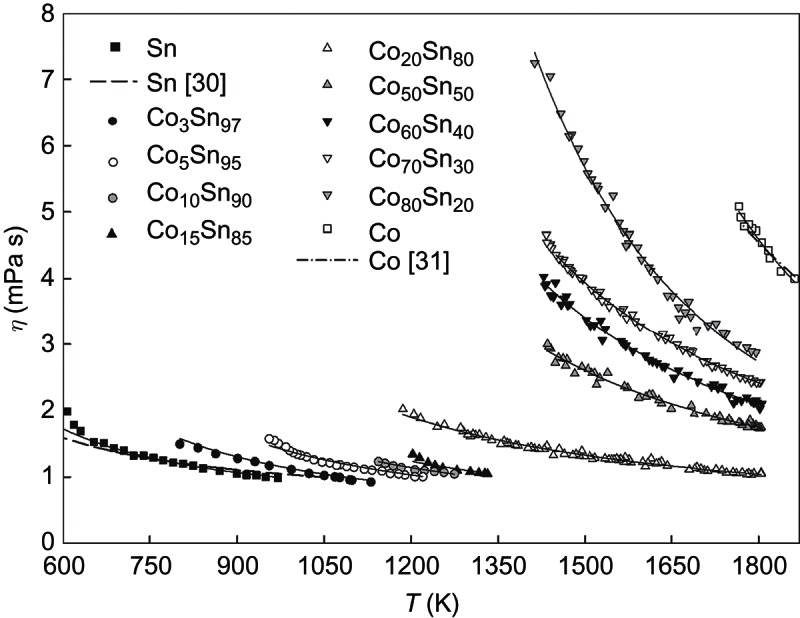
The temperature dependence of the viscosity for liquid Co–Sn alloys.

Measurements were performed for binary alloys with compositions Co_80_Sn_20_, Co_70_Sn_30_, Co_60_Sn_40_, Co_50_Sn_50_, Co_20_Sn_80_, Co_15_Sn_85_, Co_10_Sn_90_, Co_5_Sn_95_ and Co_3_Sn_97_ (in at. %). We choose these compositions because they are close to the compositions of the chemical compounds and/or the eutectic point. The temperature dependencies of the viscosities of liquid Co–Sn alloys together with viscosity data of the pure components [30,31] are presented in Figure 1.

The viscosities increase when the temperature is lowered according to the Arrhenius-type equation:

(1) 
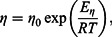

where *η*_0_ is the constant, *E_η_* is the energy of activation of viscous flow, *T* is the absolute temperature and *R* is the ideal gas constant. Such behaviour is illustrated in Figure 1, where the viscosity of the samples as a function of temperature is given.

A temperature dependence of this type is often found for the viscosity in melts of simple metals, if the properties of the melt can be well described by a hard-sphere model [32]. The lack of any deviation of the experimental viscosity from the exponential law in Co–Sn melts indicates that no significant structure transformations occur in the liquid state when approaching the solidification temperature. Values of the activation energy of viscous flow for all investigated samples were determined by least square regressional fits of the experimental data to Equation (1). These values are presented in Table 1 together with *η*_0_ data.

**Table 1.** Fitting numerical parameters of the Arrhenius-type Equation (1).

Sample, at. %	*η*_0_ (mPa s)	*E_η_* (kJ mol^−1^)
Sn	0.407	7.2
Co_3_Sn_97_	0.273	11.7
Co_5_Sn_95_	0.256	13.9
Co_10_Sn_90_	0.246	15.3
Co_15_Sn_85_	0.254	15.8
Co_20_Sn_80_	0.318	17.8
Co_50_Sn_50_	0.245	29.5
Co_60_Sn_40_	0.186	36.3
Co_70_Sn_30_	0.214	36.4
Co_80_Sn_20_	0.072	54.4
Co	0.048	68.1

**Figure 2.**
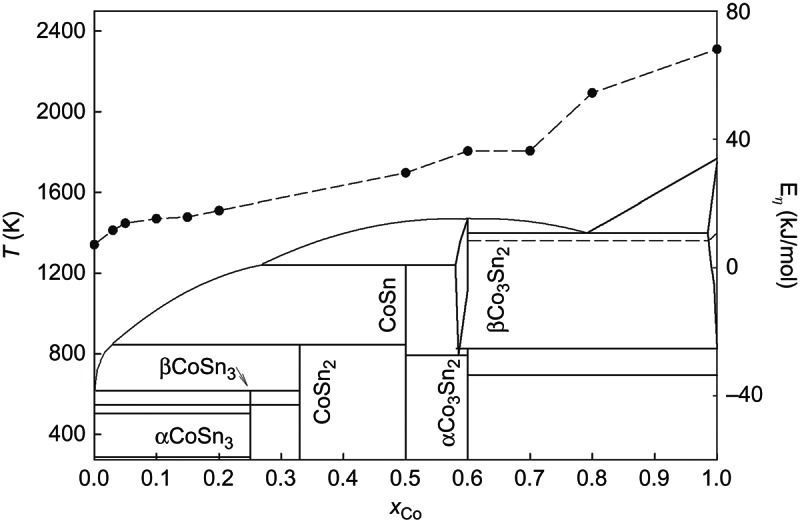
The variation of the activation energy with concentration superimposed on the phase diagram.

Figure 2 shows the variation of the activation energy with concentration superimposed on the phase diagram [7]. Clearly, a pronounced similarity between the activation energy and the liquidus curve is obvious, with the exception of the near eutectic region (80 at. % Co). According to X-ray diffraction data [33], short-range order in the liquid Co_79.5_Sn_20.5_ alloy consists of βCo_3_Sn_2_ type clusters and βCo clusters, each accounting for about 50% in the melt. Such a structure leads to nucleation difficulties and is the reason of the high value of the activation energy of viscous flow. Therefore, the obtained positive deviation of the activation energy at the eutectic point can be related to the existence of an inhomogeneous short-range order in the liquid state. As seen in Figure 2, the activation energy values obtained using two different viscometers are also in good agreement.

Several thermodynamic models and semi-empirical equations were developed to evaluate the viscosity of liquid alloys:

According to the Budai–Benkö–Kaptay equation, the viscosity of a multi-component alloy can be written as [22]

(2) 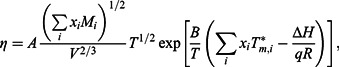

where *M_i_* and *x_i_* are the atomic mass and concentration of the given component *i,* respectively; *q* is a semi-empirical parameter equal to 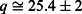 [34]; *V* is the molar volume of the alloy; Δ*Н* is the enthalpy of mixing; *А* and *B* are fitting parameters equal to (1.80 ± 0.39) × 10^−8^ (J · K^-1^ · mol^−1/3^)^1/2^ and (2.34 ± 0.20), respectively; 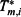 is the effective melting temperature of the component *i*:

(3) 
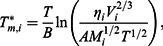

where *η_i_* and *V_i_* are the viscosity and atomic volume of the component *i*.

Singh and Sommer [35] described the viscosity of liquid metals and metal alloys using the Andrade equation [23]:

(4) 
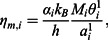

(5) 
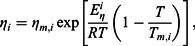

(6) 
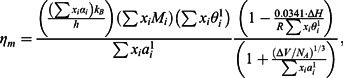

(7) 
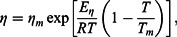

where *η_m_* and *η_m,i_* are the viscosity of the alloy and component *i* at the melting temperature *T_m_* and *T_m,i_*, respectively, *α^i^* is equal to 1.75 for normal metals and 1.35 for semimetals. The term *θ_i_* ^1^ can be determined through the relation

(8) 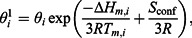

where *θ_i_* and Δ*H_m,i_* are the Debye temperature of the solid metal and the heat of fusion, *k_B_* and *h* are the Boltzmann and Planck constant, respectively. *S*_conf_ is the configurational entropy arising from disorder in the liquid state.

The Hirai equation is a semi-empirical extrapolation of the Andrade’s equation, estimating the viscosity in the following way [24]:

(9) 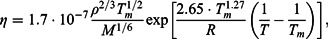

where *M* is the molar mass of the alloy calculated by the addition rule.

Recently, the following equations were proposed for the viscosity of the binary system Al-Cu by Schick et al. [25]:

(10) 
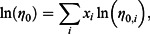

(11) 
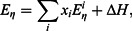

(12) 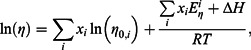

where *η_0,i_* and *E_η_^i^* are the pre-exponential factor and the activation energy of the viscous flow of the Arrhenius type equation for component *i*, respectively.

In our calculation, it was assumed that the excess volume of the alloys equals zero. The enthalpy of mixing data was taken from [13]; the density, atomic volume and configurational entropy of components were taken from [29].

**Figure 3.**
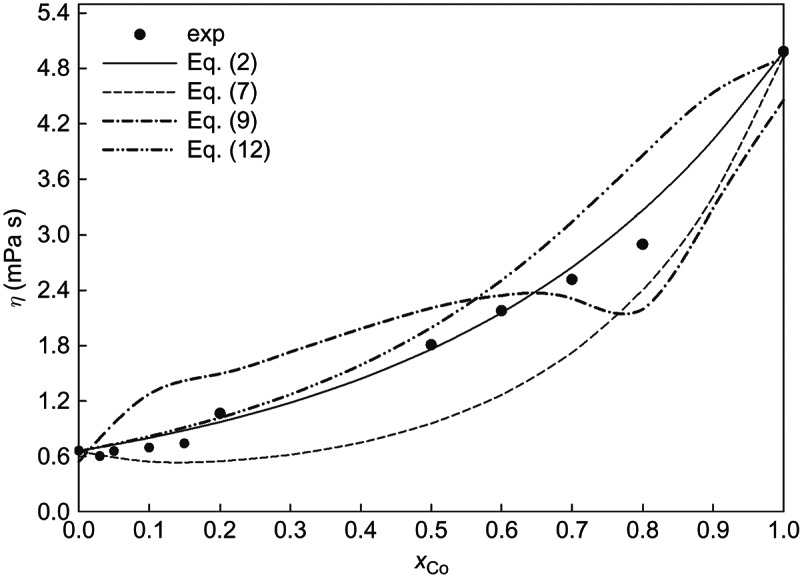
The concentration dependence of the viscosity of liquid Co–Sn at 1773 K compared with different models.

The experimental and calculated dynamic viscosities as functions of concentration for binary liquid Co–Sn alloys are presented in Figure 3. The four evaluated models, Equations (2), (7), (9) and (12), are within a band of ±0.6 mPa s around the experimental data. Hence, the agreement between the models and the data as well as among the four models is reasonable.

The semi-theoretical Andrade equations (Equations (4–7)) systematically underestimate the data. According to this approach, atoms in the liquid state at the melting temperature may be regarded as performing vibrations around their equilibrium positions with random periods and direction, like in the solid state. In other words, the melting is assumed to occur when the amplitude of atomic vibrations exceeds a certain limit, and the frequency can be calculated by Lindemann’s equation [36]:

(13) 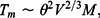

if a Debye model for the solid is assumed. In that case liquids at their melting temperatures behave like simple monoatomic liquids what is in disagreement with present-day theories of the structure of metal alloys at the melting temperature. Nevertheless, we obtained a qualitatively good correlation between experimental and calculated viscosity using Andrade`s equation.

The Hirai model (Equation (9)) underestimates the viscosity for *x*_Co _> 0.6. It overestimates it for all other concentrations. Figure 3 shows that the concentration dependence of the calculated viscosity by Hirai’s model is strikingly similar to the phase diagram. This fact is connected with the idea to relate the term corresponding to the activation energy with the melting temperature (2.65*T_m_* ^1.27^). This correlation is more evident in the region close to the melting temperature of the liquid.

The model proposed by Schick et al. (Equations (10–12)) is found to be in very good agreement with the data for cobalt mole fractions lower than 0.5. For *x*_Co_ > 0.5, however, it deviates from the experimental data, and this deviation increases with the increase of cobalt concentration. The authors of Reference [25] assumed for the calculation of the activation energy of the liquid alloys that it would be related to the free enthalpy of mixing, which requires knowledge of both, the enthalpy and the entropy of mixing. While the first can be taken from thermodynamic databases, the latter was assumed to be ideal in order to guarantee that *E*_A_, as required for the Arrhenius law, is independent of the temperature. This assumption may not always be fulfilled.

The best agreement is found for the Budai–Benkö–Kaptay approach, see Figure 3. Their model is focused on the cohesion energy in the pure liquid constituents and the enthalpy of mixing of the investigated liquid alloy using a semi-empirical factor. This factor was estimated by testing many alloys. Therefore, this model has been very successful for the description of the concentration dependence of experimental viscosity data [37].

It should be noted that because of the absence of molar volume data for liquid Co–Sn alloys we assumed in our calculations that the excess molar volume is equal to zero. It is also assumed that the entropy of mixing is ideal. Possibly, the calculations with experimental entropy of mixing and molar volume data would give a more realistic viscosity behaviour and would improve the agreement between experimental viscosity values and calculated ones using certain models.

## Conclusion

The viscosity of the binary Co_80_Sn_20_, Co_70_Sn_30_, Co_60_Sn_40_, Co_50_Sn_50_ and Co_20_Sn_80_ alloys (at. %), which correspond to the chemical compounds as well as to the eutectic and peritectic points has been studied in a wide temperature range. The correlation between the activation energy curve and the liquidus curve of the Co–Sn phase diagram suggests a correlation between interatomic interactions in the liquid and solid states.
